# Influence of Horizontal Intraoral Scan Bodies on the Trueness of Digital Impressions for Complete‐Arch Prostheses on Four Implants: An In Vitro Evaluation

**DOI:** 10.1111/clr.70001

**Published:** 2025-07-18

**Authors:** Andrea Laureti, Tiago Marques, João Pitta, Vincent Fehmer, Irena Sailer, Alessandro Pozzi, Luís Azevedo

**Affiliations:** ^1^ Department of Chemical Science and Technologies University of Rome Tor Vergata Rome Italy; ^2^ Division of Fixed Prosthodontics and Biomaterials University Clinics for Dental Medicine, University of Geneva Geneva Switzerland; ^3^ Faculty of Dental Medicine The Catholic University of Portugal (UCP) Viseu Portugal; ^4^ Center for Interdisciplinary Research in Health The Catholic University of Portugal (UCP) Viseu Portugal; ^5^ Department of Clinical Science and Translational Medicine University of Rome Tor Vergata Rome Italy; ^6^ Department of Restorative Sciences The Dental College of Georgia at Augusta University Augusta Georgia USA; ^7^ Department of Periodontics and Oral Medicine University of Michigan School of Dentistry, University of Michigan Ann Harbor Michigan USA; ^8^ Department of Restorative Dentistry and Biomaterials Sciences Harvard School of Dental Medicine Boston Massachusetts USA

**Keywords:** complete arch, digital impression, horizontal scan bodies, intraoral scanner

## Abstract

**Objective:**

To assess the impact of horizontal intraoral scan bodies (H‐ISBs) on the trueness of complete‐arch digital impressions compared to vertical ISBs (V‐ISBs). To evaluate trueness among four intraoral scanners (IOS) and inter‐operator variability across different ISB × IOS combinations.

**Materials and Methods:**

Digital impressions were made from a dental cast with four multi‐unit analogs using four H‐ISBs: H‐NB, H‐NS, H‐M6, H‐SF, and a V‐ISB (V‐EA) as a comparison. Two operators performed 10 scans per ISB with four IOS devices (i5D, PS, T3, T4), generating 400 impressions. Reference scans were obtained with a desktop scanner, and trueness was analyzed using root‐mean‐square (RMS) error calculations (*α* = 0.05).

**Results:**

H‐NS and H‐SF exhibited the highest trueness across IOSs, whereas H‐NB and H‐M6 showed lower trueness. V‐EA outperformed H‐NB and H‐M6 but not H‐NS and H‐SF. Significant IOS‐ISB interaction effects (*p* < 0.01) indicated H‐SF as the most accurate, especially with PS. T4 and i5D displayed greater variability, particularly with H‐NB. V‐ISBs exhibited higher inter‐operator variability compared to H‐ISBs.

**Conclusions:**

H‐ISBs did not perform better than V‐ISBs in all scenarios. The interactions among ISB design, IOS type, and operator significantly affect the digital impression trueness. The discrepancies measured among the systems remain well below the currently accepted threshold for clinically relevant misfit, supporting the suitability of the horizontal configuration for complete‐arch impressions.

## 1 Introduction

The shift from conventional to digital impressions in implant dentistry has significantly improved efficiency, optimized clinical procedures, and enhanced prosthesis fabrication (Joda and Brägger 2016; Joda et al. 2017). However, digital impressions present specific challenges, particularly in complete‐arch restorations, where capturing accurate data is crucial (Wulfman et al. 2020; Papaspyridakos et al. 2020).

Conventional impression techniques involve multiple steps, increasing the risk of inaccuracies (Pozzi et al. 2013). Intraoral optical scanning (IOS) provides a reliable alternative for capturing implant positions, but its accuracy depends on several patient or operator‐related factors, such as consistent reference points along the dental arch (Revilla‐León, Kois, and Kois 2023a, 2023b). In completely edentulous patients, the absence of stable anatomical landmarks complicates image alignment, often leading to stitching errors in three‐dimensional (3D) image superimposition (Gómez‐Polo, Sallorenzo, et al. 2024). Additionally, the vertical orientation of traditional intraoral scan bodies (ISBs) can increase the risk of misalignment across the arch (Park et al. 2024).

To address these challenges, horizontal ISBs (H‐ISBs) were introduced, offering a more stable and efficient scanning approach, particularly for edentulous cases (Giglio et al. 2024; Klein et al. 2023). Unlike vertical ISBs (V‐ISBs), which require complex scanning paths, H‐ISBs create stable geometric references, reducing image overlap and stitching errors (Giglio et al. 2024). Additionally, H‐ISBs can bridge inter‐implant spaces more effectively, eliminating the need for additional techniques such as splinting ISBs or adding artificial landmarks (Retana et al. 2023; Pozzi et al. 2022; Azevedo et al. 2024b). These advantages make H‐ISBs a promising option for complete‐arch implant digital impressions, particularly in edentulous patients, by reducing scan time and improving ease of use (Ashraf et al. 2023).

Several studies have evaluated the impact of ISB material, geometry, and height on digital impression accuracy (Mizumoto et al. 2020; Gómez‐Polo et al. 2023; Azevedo et al. 2024a). Understanding these factors helps clinicians select the best ISB design for their specific IOS system, optimizing accuracy in the digital workflow.

Despite these potential advantages of H‐ISBs, to the author's knowledge, no studies have directly compared the trueness of H‐ISBs and V‐ISBs across different IOS systems. This study aims to fill this gap by evaluating the trueness of H‐ISBs in complete‐arch implant digital impressions. The primary outcome is to assess the impact of H‐ISBs on the trueness of complete‐arch implant digital impressions for four‐implant complete‐arch cases and compare them with V‐ISBs. The secondary outcome is to evaluate the influence of different IOS devices on trueness and examine inter‐operator variability across different ISB × IOS combinations. The null hypothesis stated that no significant differences would be found in trueness between vertical and horizontal ISBs, among different IOSs, or between operators.

## 2 Materials and Methods

A definitive poured dental cast of an edentulous mandible with a soft‐tissue replica was created and used as the reference dental cast. Four multi‐unit analogs (Nobel Biocare, Switzerland) were positioned in the mandibular model at specific sites (second premolars, and lateral incisors), 3 mm below the model's surface. The study did not require ethical approval, as it involved no human participants or patient data.

**FIGURE 1:**
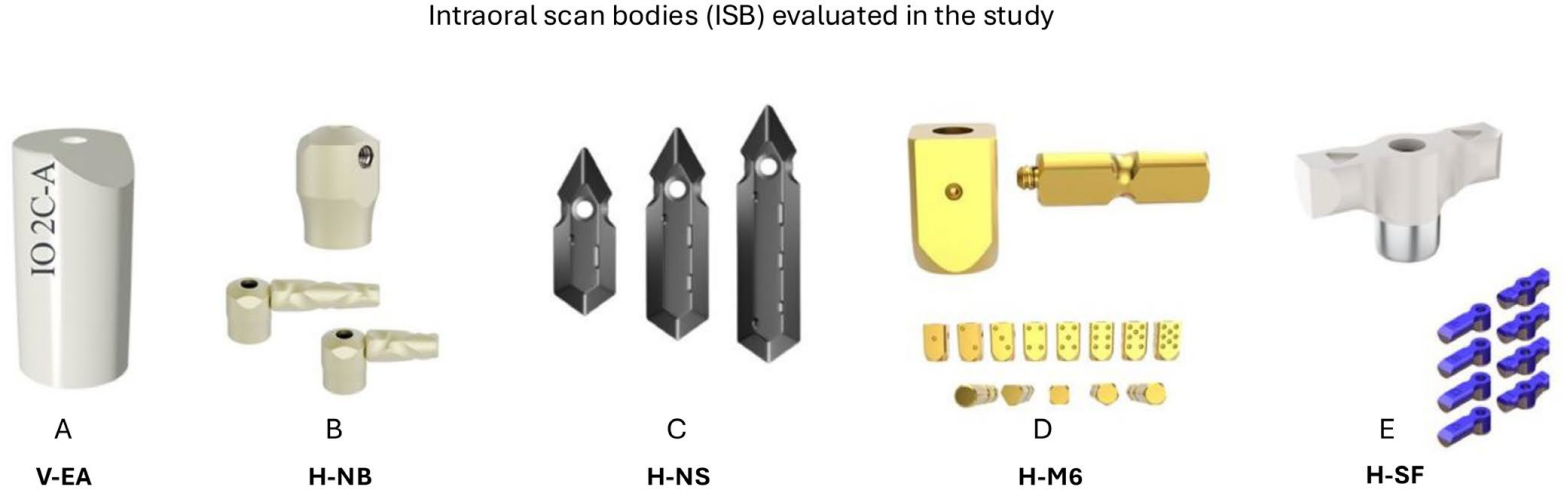
Intraoral scan bodies (ISBs) of various designs (vertical and horizontal) from different manufacturers, included in the study; (A) V‐EA (Elos Accurate, Nobel Biocare); (B) H‐NB (Multi‐unit Polo, Nobel Biocare); (C) H‐NS (Nexus Scan Gauges, Nexus iOS); (D) H‐M6 (M6 Dental, MY Digital Implant); (E) H‐SF (SmartFlags, Apollo).

Four H‐ISBs (H‐NB: Nobel Biocare Multi‐unit Polo, H‐NS: Nexus Scan Gauges, H‐M6: M6 Dental Multi‐unit abutment, and H‐SF: Apollo SmartFlags) were selected, alongside one V‐ISB (V‐EA: Elos Accurate, Nobel Biocare, Switzerland) as the standard design (Figure 1). The H‐ISBs differed in material and structure: H‐NB, H‐NS, and H‐M6 were entirely composed of titanium, with H‐NB and H‐M6 featuring a two‐piece design with a horizontal component that screws into the main body, whereas H‐NS was a single‐piece design. H‐SF combined a medical polymer (PEEK) with an anti‐reflective surface to facilitate scanning, whereas its titanium base ensured a tight connection with the implant. The V‐ISB (V‐EA) was a one‐piece structure composed of PEEK. All ISBs were tightened to 10 Ncm using an automatic torque device (Endo‐mate TC2, NSK, Japan) and were not moved between registrations to avoid positional errors. Visual inspection confirmed that each ISB adhered to the manufacturer's specifications regarding placement along the arch before scanning.

A high‐accuracy desktop scanner (IScan4D LS3i, Imetric 4D, Switzerland), properly calibrated before use, was used to scan the reference dental cast, generating the reference digital cast (RDC) with an accuracy of ±5 μm, per manufacturer specifications (Doukantzi et al. 2021; Marchand et al. 2022). For each ISB type, a reference dental cast was obtained and scanned to establish an accurate baseline for comparison. The resulting scan was saved as a standard tessellation language file (STL) to serve as the baseline for comparisons.

Four IOSs were used for the digital test scans: TRIOS 3 (T3) and TRIOS 4 (T4) (3Shape, Denmark), iTero Element 5D (i5D) (Align Technology, USA), and Primescan (PS) (Dentsply Sirona, USA). A standardized zigzag scan technique was employed for all devices, beginning from the most distal ISB of the fourth quadrant, as recommended in previous studies (Gómez‐Polo, Cascos, et al. 2024; Li et al. 2022). Two experienced operators (L.A. and A.L.) conducted the scans within 1 month (November–December 2023) after completing three training sessions of 20 min with each IOS. The scans were performed using the most recent acquisition software available for each IOS at the time of the study. Settings for resolution, depth, and scanning angle were standardized across all devices.

**FIGURE 2:**
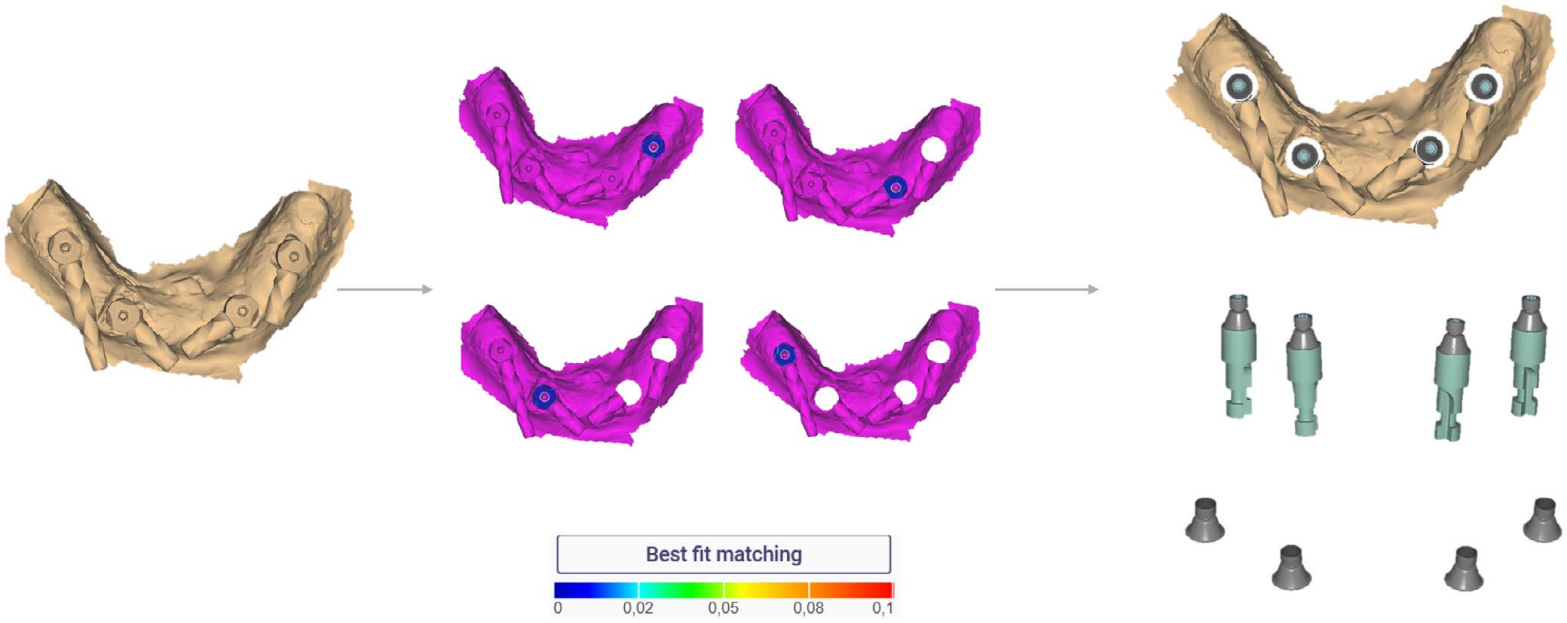
Digital workflow for aligning STL files from intraoral and desktop scanners with the CAD library's scan body (ISB) using exocad software (exocad DentalCAD, exocad GmbH), standardizing the multi‐unit abutment for 3D evaluation and comparison.

A formal power analysis was not conducted for this study. However, the sample size was determined on the basis of previous research from the authors on digital complete‐arch implant impressions, which used similar methodologies (Azevedo et al. 2024a, 2024b). In a prior study, a power analysis indicated that a minimum sample size of *n* = 6 per group was required for adequate statistical power (95%) when comparing different scanning techniques. To maintain consistency with previous studies and allow for potential variability, a sample size of *n* = 10 per group was selected. This approach aligns with established research in the field and has been used in similar trueness evaluations of IOSs and ISBs (Amin et al. 2017; Mangano et al. 2019, 2020; Papaspyridakos et al. 2016). On the basis of this, for each ISB type and IOS, 10 scans were made of the RDC (*n* = 10). Ambient light, room temperature, and humidity were controlled to ensure optimal conditions for scanning (Revilla‐León et al. 2020; Agustín‐Panadero et al. 2023; Revilla‐León, Gohil, et al. 2023). All digital scans were acquired under a constant room temperature of 20°C in a room with no external daylight. The ceiling fixture light was turned on during scanning. Although relative humidity was not specifically measured, all scans were performed under the same environmental conditions to minimize potential variability. Studies have shown that high humidity levels can affect scanning accuracy, time, and the number of photograms, reinforcing the importance of maintaining a stable scanning environment (Agustín‐Panadero et al. 2023). The scanning sequence was randomized to minimize bias using spreadsheet software (Excel; Microsoft Corp). The resulting STL files were aligned and compared with CAD software (exocad DentalCAD 3.1 Rijeka; exocad GmbH, Germany) using the ISB library files, with the same multi‐unit abutment analog for all files to facilitate 3D evaluation and comparison (Figure 2). All alignments with the corresponding library's ISB were performed by the same person (L.A.), who had experience working with CAD software.

**FIGURE 3:**
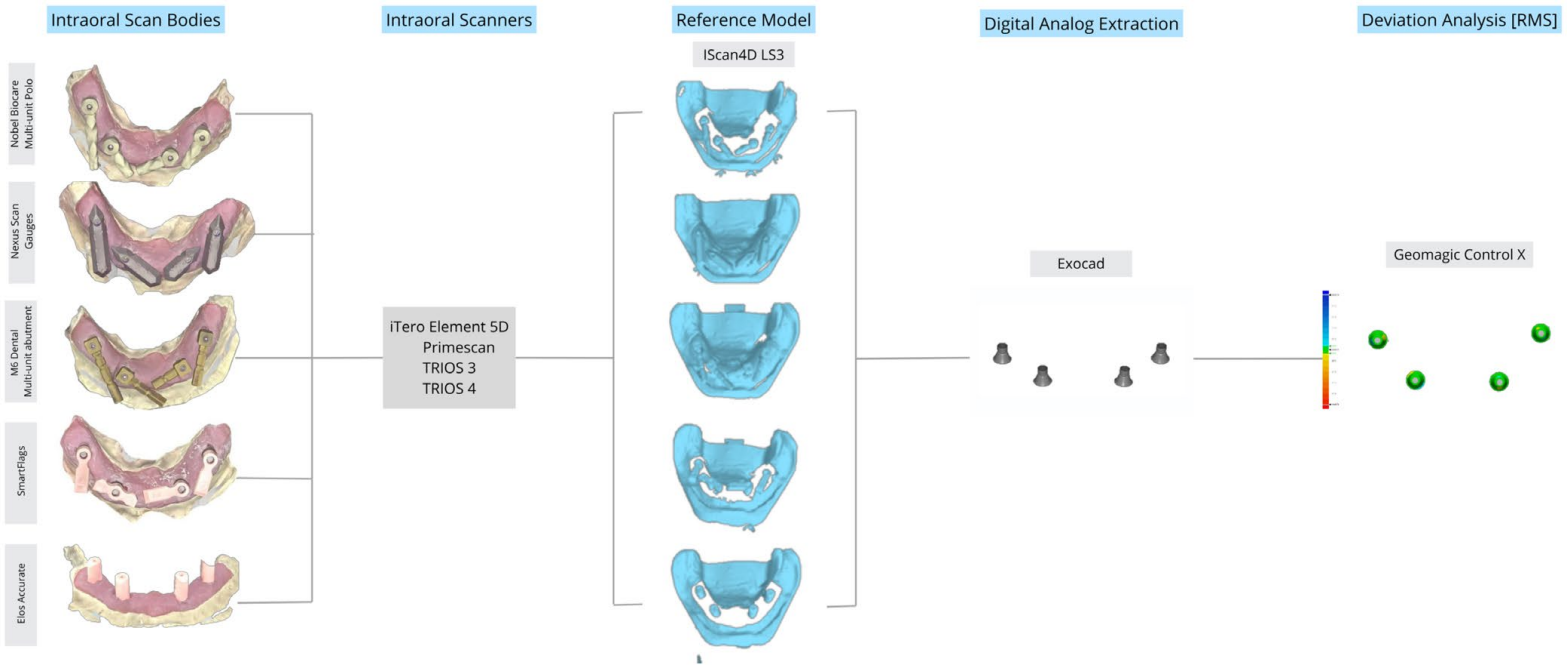
Summary of the 3D digital analysis for the superimposition of scans according to the “Best‐fit Alignment” method in Geomagic Control X.

Metrology software (Geomagic Control X 2022.1; 3D Systems Inc., USA) was used to analyze trueness by comparing digital analogs (digital multi‐unit abutments) to the RDC (reference). To ensure precise alignment, the STL files were first aligned using the ‘Align Between Measured Data’ function, followed by ‘Best‐Fit Alignment’ for optimal superimposition. Deviations were then analyzed with the ‘3D Compare’ function, producing a color map that visualized the discrepancies between the models, with a tolerance range of ±0.02 mm and deviation limits spanning from +0.3 mm to −0.3 mm (Figure 3). The mean root‐mean‐square (RMS) error was calculated to quantify the overall deviation, using the square root of the average squared differences between corresponding points on the two digital multi‐unit abutments. RMS is a mathematical measure used to quantify the extent of variation within a dataset.

The primary outcome measured was the trueness of each ISB type, recorded in microns (μm). Trueness evaluated how close the digitized test object (ISBs) was to the true dimensions of the RDC.

Descriptive statistics, including the median, minimum, and maximum values, were calculated. To account for data correlation, an initial model with a random intercept was fit. However, a likelihood ratio test showed no significant difference compared to the uncorrelated model (*p* = 0.71), and the covariance was near zero (−4.64 × 10^−8^), indicating that repeated measurements were not correlated. As a result, a full factorial three‐way ANOVA without random effects was selected. In this model, RMS was the dependent variable, with operator, IOS, ISB, and their interactions as independent variables. Wald tests were performed to examine the main effects and interactions, and a Tukey test assessed group differences. Trueness was defined as the mean 3D deviation between corresponding ISBs in the test scan and the RDC. Statistical significance was set at *p* < 0.05, and all analyses were conducted using Stata 18.0 (StataCorp, USA).

## 3 Results

Descriptive statistics are summarized in Table 1. The histogram indicated a distribution close to normal for the outcome variable. Although the Shapiro–Wilk test yielded *p* < 0.05, given the large sample size (*n* = 400), the histogram was considered a more reliable indicator of normality.

**TABLE 1:** Median, minimum, and maximum mean root‐mean‐square (RMS) values of different intraoral scan bodies (ISBs) for each intraoral scanner (IOS) (μm).

		iTero Element 5D	Primescan	TRIOS 3	TRIOS 4
		Median (min–max)	Median (min–max)	Median (min–max)	Median (min–max)
Operator 1	V‐EA	29 (23–45)^a; α^	18 (13–28)^a; α,β^	11 (6–19)^a; β,γ^	28 (23–41)^a; α,β,γ^
	H‐NB	37 (33–45)^a,b; α^	14 (8–22)^a,b; β^	31 (21–46)^b; α,γ^	36 (31–66)^a,b; α,γ^
	H‐NS	11 (7–17)^c; α^	19 (15–23)^a,b,c; α,β^	16 (10–26)^a,c; α,β,γ^	40 (20–43)^a,b,c^
	H‐M6	49 (35–72)^b,d; α^	16 (8–16)^a,b,c; β^	24 (16–35)^b,c; β,γ^	36 (24–61)^a,b,c,d; α^
	H‐SF	29 (24–38)^a,b; α^	6 (4–15)^b,c; β^	14 (9–19)^a,c; β,γ^	22 (14–36)^a,c; α,γ^
Operator 2	V‐EA	15 (11–44)^a; α^	31 (18–44)^a; α,β^	28 (20–34)^a; α,β,γ^	18 (15–30)^a; α,β,γ^
	H‐NB	37 (30–49)^b; α^	20 (17–26)^a,b; β^	27 (23–55)^a,b; α,β,γ^	46 (34–62)^b; α^
	H‐NS	14 (2–17)^a,c; α^	13 (9–14)^b,c: α,β^	15 (9–18)^c; α,β,γ^	21 (3–22)^a,c; α,β,γ^
	H‐M6	34 (23–49)^b; α^	13 (9–21)^b,c; β^	18 (12–31)^a,c; β,γ^	40 (31–65)^b,d; α^
	H‐SF	34 (29–42)^b; α^	8 (6–16)^b,c; β^	16 (13–23)^a,c; β,γ^	25 (12–46)^a,c; α,γ^

*Note:* Values sharing the same letter within a column (a, b, c and d) and within a row (α, β and γ), for each operator, are not statistically different at the 5% level. Tukey adjustment applied for multiple comparisons.

For ISB comparisons regardless of IOS, in general, V‐EA demonstrated significantly lower RMS values (higher trueness) compared to H‐NB and H‐M6 (*p* < 0.01). Among H‐ISBs, H‐SF consistently showed the lowest RMS values, with significant differences observed between several H‐ISB configurations (Table 1).

For IOS comparisons, in general, Primescan achieved the highest trueness across all ISBs, particularly with H‐SF, whereas iTero Element 5D showed the lowest trueness for H‐NB and H‐M6. TRIOS 3 and TRIOS 4 exhibited moderate trueness, though TRIOS 4 displayed greater variability, particularly for H‐NB. Detailed IOS comparisons are reported in Table 1.

**FIGURE 4:**
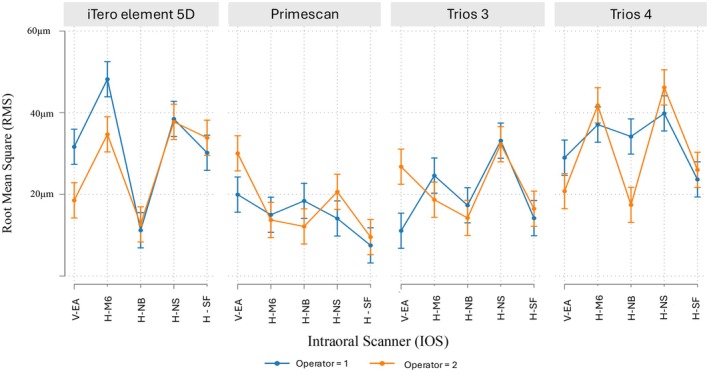
Margin plots for mean root‐mean‐square (RMS) by operator, intraoral scanner (IOS), and intraoral scan body (ISB) with 95% confidence intervals. Non‐overlapping confidence intervals indicate statistically significant differences between operators for specific ISB × IOS combinations.

Figure 4 illustrates operator differences across specific ISB × IOS combinations, with non‐overlapping confidence intervals indicating statistically significant discrepancies. Notably, significant differences were observed for iTero Element 5D (*p* = 0.02), Primescan (*p* = 0.04), and TRIOS 3 (*p* < 0.01) when used with V‐EA, as well as for iTero Element 5D with H‐M6 (*p* = 0.01) and TRIOS 4 with H‐NS (*p* < 0.01).

**FIGURE 5:**
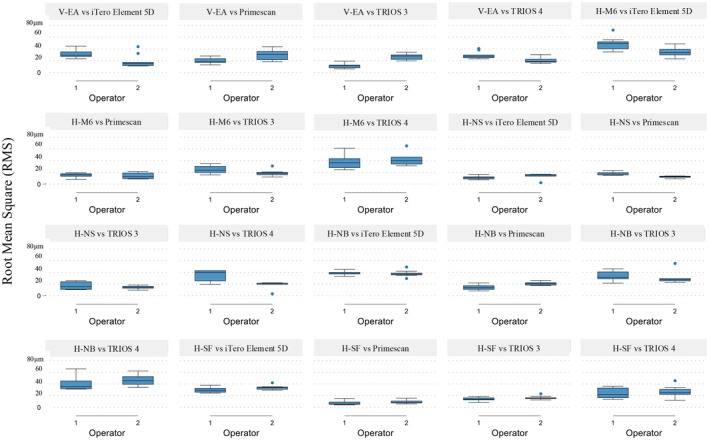
Boxplots of the mean root‐mean‐square (RMS) by intraoral scanner (IOS), intraoral scan body (ISB) and operator.

**FIGURE 6:**
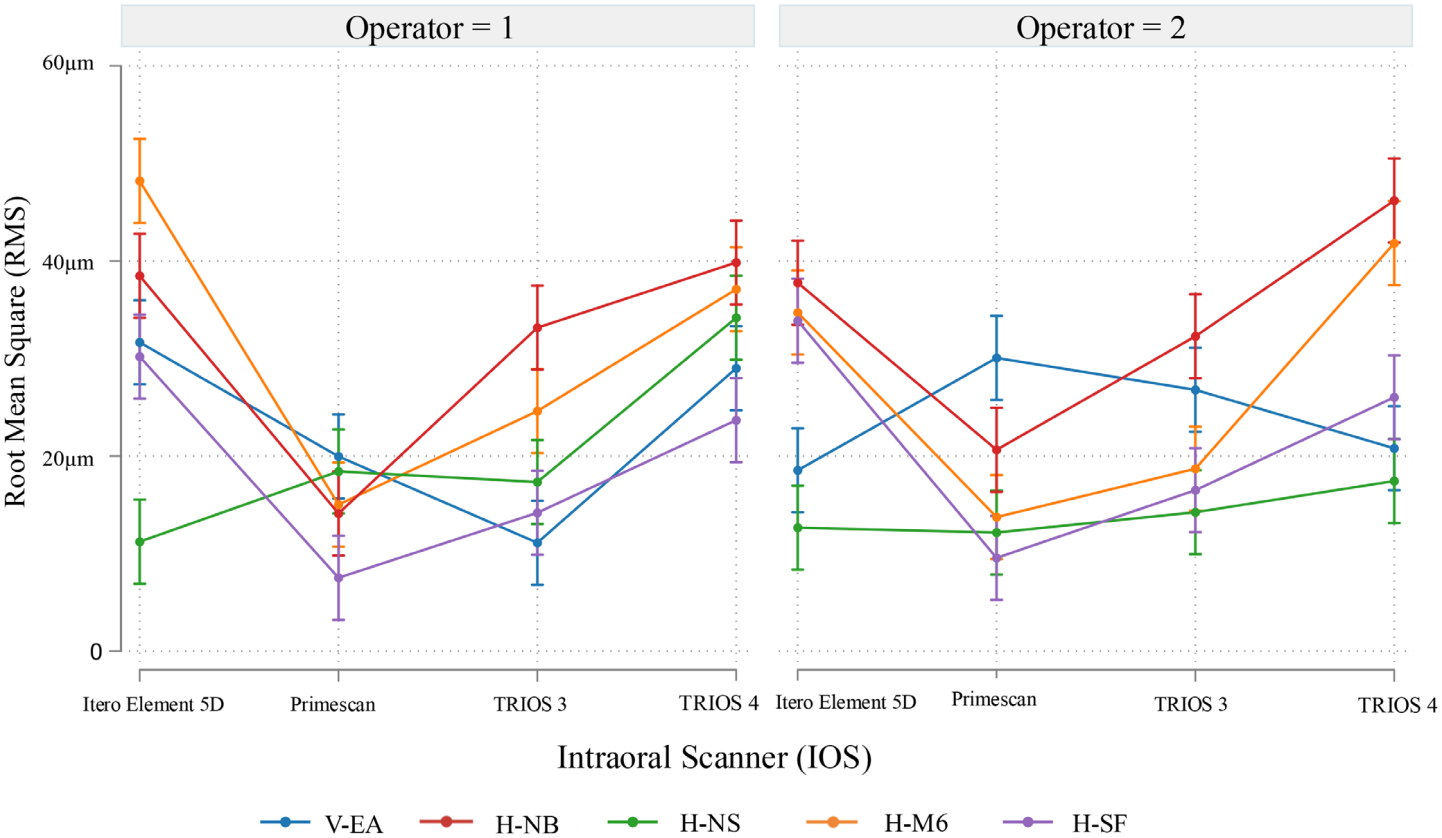
Margin plots for mean root‐mean‐square (RMS) by operator, intraoral scanner (IOS) and intraoral scan body (ISB) with 95% confidence intervals.

Regression analysis (Table 2) confirmed that both IOS and ISB significantly influenced RMS values (*p* < 0.01), with pairings such as Primescan with H‐SF achieving the highest trueness, whereas iTero with H‐NB and H‐M6 showed the most variability. Figures 5 and 6 visually compare RMS distributions, highlighting Primescan's consistent trueness, particularly with H‐SF, and iTero Element 5D's variability with H‐NB and H‐M6.

**TABLE 2:** Contrasts for main effects and interaction terms (Wald tests).

	Degrees of freedom	*F*	*p*
Operator	1	1.10	0.295
ISB	4	68.49	< 0.001
IOS	3	111.99	< 0.001
Operator × IOS interaction	3	5.30	< 0.001
Operator × ISB interaction	4	7.01	< 0.001
IOS × ISB interaction	12	19.23	< 0.001
Operator × IOS × ISB interaction	12	7.30	< 0.001

## 4 Discussion

The findings of this study underscore the significant influence of ISB design, IOS selection, and ISB‐IOS interactions on the trueness of complete‐arch digital impressions. Consistent with prior research (Gómez‐Polo et al. 2023; Azevedo et al. 2024a), IOS type and ISB design and material were key factors influencing trueness, as evidenced by significant differences in RMS values among IOS‐ISB combinations. H‐ISBs, particularly H‐NS and H‐SF, generally achieved higher trueness across IOSs compared to V‐EA. However, in certain cases, V‐EA outperformed H‐NB and H‐M6, an unexpected finding. Therefore, the null hypothesis was partially rejected, suggesting that horizontal configurations do not universally provide the highest trueness across all IOSs and scenarios.

One potential explanation for V‐ISB's higher trueness with certain IOSs, such as the iTero Element 5D, may lie in the compatibility between the scanner's optical technology and the V‐ISB's design. Unlike H‐ISBs, which were developed to simplify the scanning path (Giglio et al. 2024), V‐ISBs align with traditional scanning protocols that may reduce image misalignment by enabling the IOS to collect consistent vertical data points. In this study, the iTero Element 5D showed significant variability with certain H‐ISBs, particularly H‐NB and H‐M6. The better performance of iTero with some V‐ISB compared to H‐ISBs may be attributed to the specific design and material compatibility between the IOS and ISB. Notably, the lowest trueness of the iTero Element 5D was specifically associated with H‐NB and H‐M6, which share a similar structural design. Unlike H‐NS and H‐SF, which have a single, integrated fixture, H‐NB, and H‐M6 include a horizontal component that must be screwed into the main body. This design feature may impact trueness and warrants further investigation. Additionally, the zigzag scanning technique, adopted by the authors, may have exacerbated image stitching challenges, particularly with wider H‐ISBs, leading to alignment issues that were less prominent in V‐ISB scans. Previous research has shown that scan pattern significantly influences the accuracy and speed of complete‐arch digital implant impressions (Li et al. 2022; Pattamavilai and Ongthiemsak 2024). Similarly, a clinical study by Gómez‐Polo, Cascos, et al. (2024) found that scanning patterns impact not only accuracy but also scan time and the number of photograms captured. The authors recommended the zigzag and O‐Lock scanning patterns for complete‐arch implant scans when using specific IOSs (Gómez‐Polo, Cascos, et al. 2024). Given these findings, further investigation is needed to determine the most suitable scanning strategies for H‐ISBs, ensuring optimal trueness and efficiency in complete‐arch digital impressions.

The Primescan consistently demonstrated higher trueness across ISBs, particularly with H‐NS and H‐SF, confirming previous studies' outcomes on its scanning capabilities (Azevedo et al. 2024b; Ashraf et al. 2023). Notably, Primescan's trueness was significantly higher with H‐SF compared to V‐EA, supporting the notion that horizontal configurations leverage Primescan's advanced capabilities, enabling enhanced data capture across the complete arch and minimizing stitching errors.

Conversely, the TRIOS systems exhibited variable performance, with TRIOS 3 delivering more consistent results across ISBs compared to TRIOS 4, which showed higher RMS values. This finding aligns with previous studies that reported lower trueness for the TRIOS 4 (Azevedo et al. 2024a). Additionally, both TRIOS 3 and TRIOS 4 demonstrated lower trueness with H‐NB and H‐M6, a trend previously noted with the iTero Element 5D system.

Operator differences were only observed for specific ISB × IOS combinations. This underscores that although operator skill influences impression accuracy (Zarauz et al. 2023), according to different systematic reviews, the choice of IOS and ISB has a greater impact (Gómez‐Polo et al. 2023; Gehrke et al. 2024). However, the results revealed that with the majority of IOS, the V‐EA exhibited significant differences between operators. Despite V‐EA generally demonstrating higher trueness compared to H‐NB and H‐M6, it also introduced greater variability in operator performance. As observed in Table 1, Operator 2 achieved better results with iTero Element 5D and TRIOS 4, whereas Operator 1 performed better with Primescan and TRIOS 3. This suggests that even among experienced operators, V‐ISBs may lead to greater deviations, potentially because of their design or scanning requirements. Conversely, H‐ISBs demonstrated more consistent performance between operators, suggesting that their structure or scanning process may reduce inter‐operator variability. The regression analysis confirmed significant IOS‐ISB interactions, highlighting the importance of selecting compatible IOS‐ISB pairs to achieve high trueness. For instance, Primescan consistently achieved the highest trueness when paired with H‐SF, whereas the performance of the iTero Element 5D was notably influenced by the specific ISB type.

The authors emphasize that although the differences in trueness observed in this study are statistically significant, they are unlikely to be clinically relevant. This is because the currently accepted threshold for misfits is generally considered to range between 100 and 150 μm, as reported by several authors (Jemt and Book 1996; Jemt and Lie 1995).

Limitations of this study include its in vitro experimental design, which does not fully replicate clinical conditions such as patient movement, saliva, and soft tissue interactions, all of which may influence scanning accuracy. Additionally, the standardized zigzag scanning protocol, although commonly used, may not be the most optimal technique for all IOS and ISB combinations. Variations in IOS technologies could have also impacted the results, as each scanner uses different optical scanning principles and data processing algorithms. Furthermore, a limitation of this study is the absence of repeated measurements by multiple raters, which prevents the calculation of Intraclass Correlation Coefficient (ICC) to quantify inter‐rater reliability. Future research should incorporate duplicate measurements to assess rater variability and allow for potential recalibration if ICC values indicate low agreement.

The measurement method employed in this study, root mean square (RMS), has been widely used in comparable research to assess deviations in implant scans (Çakmak et al. 2020; Gómez‐Polo, Cimolai, et al. 2024; Kanjanasavitree et al. 2022). Cakmak et al. (2022), in their study of the effects of 3D analysis software and operator variability on scan deviations, found that all tested software systems produced similar results, with the exception of one, regardless of the operator involved. Furthermore, the inter‐operator reliability across the tested 3D analysis software was found to be generally high (Cakmak et al. 2022). These findings suggest that the choice of evaluation software has minimal impact on the measurement outcomes, highlighting that various software systems and different operators can yield comparable results when measuring deviations in implant scans.

Future research should prioritize in vivo assessments to determine whether the advantages of specific H‐ISBs are maintained under clinical conditions. Further investigations should also explore how different scanning patterns and IOS technologies interact with various ISB designs and materials to affect trueness. IOS developers could enhance software algorithms by integrating the virtual geometry of ISBs. With the addition of artificial intelligence‐assisted scanning, the IOS could match the ISB to its virtual geometry during impression taking, potentially improving accuracy. Implementing these findings into clinical practice, alongside appropriate training, could optimize digital workflows in implantology, ultimately improving clinical outcomes for edentulous complete‐arch restorations. These considerations emphasize the importance of understanding the technologies involved and recognizing the limitations of each IOS in detecting different ISB designs or materials. This awareness will help guide the selection of the optimal IOS and ISB combination for achieving accurate digital impressions in complete‐arch restorations.

## 5 Conclusions

Within the limitations of this study, two out of four horizontal scan bodies showed significantly higher trueness across different intraoral scanners than the vertical scan body, with RMS values ranging between 2 and 43 (H‐NS) and 4 and 46 (H‐SF) microns. The maximum deviation experienced was 72 μm, which remains well below the currently accepted threshold for clinically relevant misfit, underscoring the favorable effect of the horizontal configuration on the trueness of complete‐arch digital impression. All interactions among intraoral scanner type, operator influence, and scan body design were statistically meaningful, highlighting the complexity of the complete‐arch scenario and the potential contribution of each variable to impression trueness.

## Author Contributions

**Andrea Laureti:** conceptualization, investigation, writing – original draft. **Tiago Marques:** software, data curation, supervision. **João Pitta:** supervision, writing – review and editing, methodology, validation. **Vincent Fehmer:** funding acquisition, writing – review and editing, project administration, supervision, resources. **Irena Sailer:** resources, supervision, writing – review and editing, funding acquisition, methodology, project administration. **Alessandro Pozzi:** writing – review and editing, supervision, validation. **Luís Azevedo:** conceptualization, investigation, funding acquisition, methodology, writing – original draft, software, project administration, data curation, resources.

## Conflicts of Interest

The authors declare no conflicts of interest.

## Data Availability

The data that support the findings of this study are available from the corresponding author upon reasonable request.
